# A novel pathway to produce butanol and isobutanol in *Saccharomyces cerevisiae*

**DOI:** 10.1186/1754-6834-6-68

**Published:** 2013-05-04

**Authors:** Paola Branduardi, Valeria Longo, Nadia Maria Berterame, Giorgia Rossi, Danilo Porro

**Affiliations:** 1 University of Milano Bicocca, Piazza della Scienza 2, Milano, 20126, Italy; 2 Current address: PTA (Schweiz) GmbH, Hohlstrasse 192, Zürich, CH-8004, Switzerland

**Keywords:** Butanol, Isobutanol, Glycine, *Saccharomyces cerevisiae*

## Abstract

**Background:**

The sustainable production of biofuels remains one of the major issues of the upcoming years. Among the number of most desirable molecules to be produced, butanol and isobutanol deserve a prominent place. They have superior liquid-fuel features in respect to ethanol. Particularly, butanol has similar properties to gasoline and thus it has the potential to be used as a substitute for gasoline in currently running engines. *Clostridia* are recognized as natural and good butanol producers and are employed in the industrial-scale production of solvents. Due to their complex metabolic characteristics and to the difficulty of performing genetic manipulations, in recent years the *Clostridia* butanol pathway was expressed in other microorganisms such as *Escherichia coli* and *Saccharomyces cerevisiae*, but in yeast the obtained results were not so promising. An alternative way for producing fusel alcohol is to exploit the degradation pathway of aminoacids released from protein hydrolysis, where proteins derive from exhausted microbial biomasses at the end of the fermentation processes.

**Results:**

It is known that wine yeasts can, at the end of the fermentation process, accumulate fusel alcohols, and butanol is among them. Despite it was quite obvious to correlate said production with aminoacid degradation, a putative native pathway was never proposed. Starting from literature data and combining information about different organisms, here we demonstrate how glycine can be the substrate for butanol and isobutanol production, individuating at least one gene encoding for the necessary activities leading to butanol accumulation. During a kinetic of growth using glycine as substrate, butanol and isobutanol accumulate in the medium up to 92 and 58 mg/L, respectively.

**Conclusions:**

Here for the first time we demonstrate an alternative metabolic pathway for butanol and isobutanol production in the yeast *S. cerevisiae*, using glycine as a substrate. Doors are now opened for a number of optimizations, also considering that starting from an aminoacid mixture as a side stream process, a fusel alcohol blend can be generated.

## Background

The production of biofuels from renewable biomasses is one of the answers to help solving the problems associated with limited fossil resources and climate changes. Butanol has superior liquid-fuel characteristics in respect to ethanol, similar properties to gasoline, and thus it has the potential to be used as a substitute for gasoline in currently running engines [1].

*Clostridia* are recognized as natural and good butanol producers and are employed in the industrial-scale production of solvents [2]. However, the complex metabolic characteristics and the difficulty of performing genetic manipulations on these bacteria fostered the exploitation of other well established cell factories. In recent years, *Escherichia coli* and *Saccharomyces cerevisiae* were engineered with the *Clostridia* butanol pathway [3,4]. While many optimizations have been successfully introduced in *E. coli*[5-8] reaching productions similar to that obtained in *Clostridia*[9], this was up to now not reported for the budding yeast. For an exhaustive view of the metabolic strategies applied for butanol and other fusel alcohol production see [10,11] and references therein. Remarkably, *Liao et al.* proposed that proteins, and thus the aminoacids released from proteins hydrolysis, can be used as a raw material for biorefining and so for biofuels production. Indeed, proteins are abundantly present as final waste of lignocellulose processing [12]. We focused our attention on the strategy which takes advantage of ketoacids as intermediates in amino acids biosynthesis and degradation metabolism to produce fusel alcohols in the yeast *S. cerevisiae*. While the pathway to isoketovalerate was better elucidated and recently successfully exploited for isobutanol formation in *S. cerevisiae*[13-17], butanol production from ketovalerate was never experimentally measured.

*S. cerevisiae* have one or more carrier systems specific for each aminoacid, even if they are not all currently known. Among them, the general aminoacids permease, encoded by *GAP*1 gene, is involved in glycine transport [18]. In the cytosol glycine can be catabolised in different ways, based on nutritional requirements. For example, it can be converted into serine through serine hydroxymethyltransferase enzyme (Shm2) [19] or into CO_2_ and NH_3_ through the enzymatic complex of glycine decarboxylase enzyme (GDC) [20].

**Figure 1:**
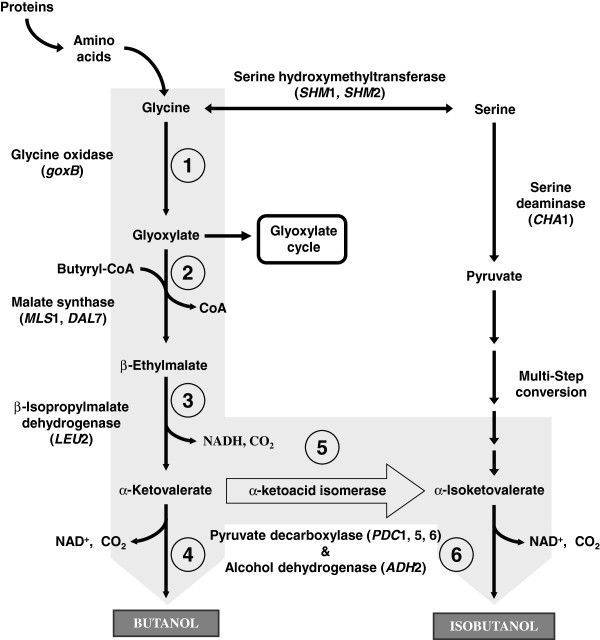
**A novel pathway for butanol and isobutanol production.** Metabolic pathway for butanol and isobutanol production from glycine in *S. cerevisiae* through the glyoxylate, β-ethylmalate and α-ketoacids intermediates (grey background arrows). The enzymatic activities involved and the associated gene(s) are also represented. Numbers inside circles indicates the steps of the pathway discussed in this study. The hypothesized isomerisation of α-ketovalerate into α-isoketovalerate is indicated by black arrow.

Villas-Bôas and co-workers [21] have *in silico* proposed the generation of glyoxylate as a consequence of glycine deamination. Moreover, the authors described the formation of α-ketovalerate and α-isoketovalerate as intermediates of the same pathway through not identified reactions. Starting from these indications and knowing from literature that α-ketovalerate can be converted into butanol [22], and that α-isoketovalerate can be converted into isobutanol [23], we cultivated yeast cells with glycine, observing the formation of both alcohols. By deeply investigating the literature, we have first (*i*) hypothesized and then (*ii*) biochemically demonstrated step by step the butanol and isobutanol production through the glycine degradation pathway via glyoxylate and α-ketoacids intermediates (Figure 1). Additionally, we suggest at least one possible gene encoding for the enzymes responsible for butanol production.

Glycine deaminase has been hypothesized to catalyze the glycine conversion into glyoxylate [21], the first step of the pathway. In *S. cerevisiae* the gene encoding for this enzyme has not been annotated yet. However, *Bacillus subtilis* gene *goxB*[24] encodes for a glycine oxidase that can catalyze this conversion (Figure 1, circle 1). For the second step, similarly to what happens in *Pseudomonas aeruginosa*[25], we hypothesized the glyoxylate condensation with butyryl-CoA to yield the β-ethylmalate intermediate (Figure 1, circle 2). β-ethylmalate might be then converted into α-ketovalerate through a β-isopropylmalate dehydrogenase enzyme (Figure 1, circle 3), as described in *E. coli*[22]. The final step is well depicted by the Ehrlich pathway: the α-ketovalerate is converted into butanol through a reductive decarboxylation reaction [26] (Figure 1, circles 4 and 6).

Summarizing, the single steps of the proposed pathway have been already described in literature, even if in different pathways and from different microorganisms: moreover, in some cases indications were provided only as enzymatic reactions. In this work we demonstrate that in the yeast *S. cerevisiae* thanks to these reactions glycine can be converted into butanol.

## Results and discussion

### Butanol and isobutanol can be obtained from glycine

All the production experiments described here and in the next paragraphs were performed in two different *S. cerevisiae* genetic background, BY and CEN.PK (see Methods for further details), proving the production of butanol and isobutanol from glycine. Here we show butanol and isobutanol kinetics and titers related to the CEN.PK background. It has to be underlined that the production levels obtained in the BY4741 background were consistent, but always lower. Because of the convenience of using single gene deletion mutants available from the Euroscarf collection, all the experiments related to the characterization of the enzymatic activities were performed using the BY4741 background.

**Figure 2:**
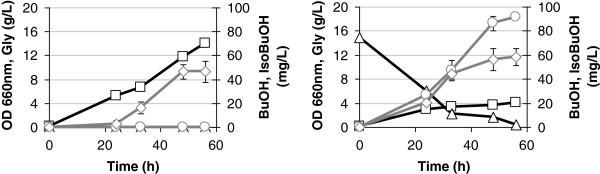
**Butanol and isobutanol accumulation from glycine.** Yeast cells were grown in Verduyn medium with ammonium sulphate (5 g/L, left panel) or glycine (15 g/L, right panel) as nitrogen source. Biomass accumulation (square), glycine consumption (triangle), butanol (circle) and isobutanol (diamond) productions are shown. The data presented here are representative of three independent experiments.

Yeast cells were grown in minimal defined medium and in the same medium but using glycine (15 g/L) instead of ammonium sulphate as nitrogen source (see Methods for details). The substitution was preferred to the addition to promote the glycine uptake, a poor nitrogen source if compared to ammonium sulphate, glutamine or glutamate [27]. Butanol and isobutanol accumulations were evaluated at different time point after the inoculum (Figure 2). We confirmed that butanol does not accumulate in minimal ammonium sulphate medium, while isobutanol does (left panel diamond symbols, 46.5 mg/L), since it can derive from other pathways (see Figure 1). When glycine is added as substrate, both butanol and isobutanol accumulate over time, reaching a registered maximum of 92 and 58 mg/L, respectively (Figure 2, right panel), together with a glycine consumption. The described experiment indicates therefore that *(i)* the butanol production requires glycine as substrate and that *(ii)* the measured isobutanol might only partially derive from the glycine degradation pathway.

In the following sections we characterize the proposed pathway step by step. The substrates used are glycine, glyoxylate, α-ketovalerate and α-isoketovalerate. Unfortunately, the intermediate β-ethylmalate is not commercially available and for this reason a coupled enzymatic reaction starting from glyoxylate as initial substrate was planned to circumvent this problem.

### The first reaction of the pathway: from glycine to glyoxylate

**Figure 3:**
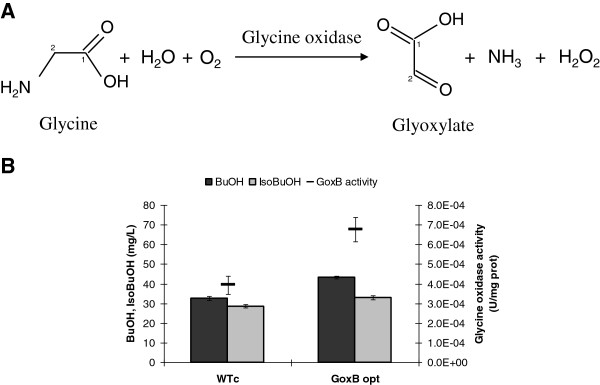
**Glycine oxidase: reaction and enzymatic activity assay.** (**A**) Reaction catalysed by glycine oxidase. The carbons of the molecules were numbered. (**B**) Glycine oxidase activity (upper bars), butanol (dark gray columns) and isobutanol (pale gray columns) for the wild type and modified yeast strains overexpressing the bacterial *goxB* gene optimized for the yeast codon usage. The data presented here are representative of three independent experiments.

The first step is the conversion of glycine to glyoxylate, by a glycine deaminase activity [21]. Since no genes are annotated in yeast encoding for this function and being the identification of a putative gene encoding for the desired function not trivial, we searched for a similar activity in other microorganisms. It has been described that in *B. subtilis* the glycine conversion into glyoxylate is catalyzed by the glycine oxidase enzyme, encoded by *goxB* gene, Figure 3A. This enzyme catalyzes the primary amines oxidative deamination to yield the corresponding α-ketoacid, with concomitant ammonium and hydrogen peroxide production. The *B. subtilis* glycine oxidase is a homotetrameric flavoprotein which effectively catalyzes the oxidation of sarcosine (N-methylglycine), N-ethylglycine and glycine. Lower activities using D-alanine, D-valine, and D-proline as substrates were described although no activities were shown on L-amino acids and other D-amino acids [24]. Consequently, the *B. subtilis goxB* gene was synthesized with an optimized codon usage (see Additional file 1) for *S. cerevisiae* (*goxB* opt) and constitutively expressed in yeast. The heterologous enzymatic activity was tested using an *in vitro* assay for glycine oxidase (as described in the Methods section [28]), Figure 3B. As expected, in the control (transformed with the empty vector) as well as in the *goxB* opt overexpressing strains the assay revealed the desired activity, being 1.5 fold higher in the recombinant yeast. It has to be mentioned that overall the measured activities are quite low: however, it might be that the assay needs to be optimized. In fact, the activity measured in total protein extract from *E. coli* BL21 strain overexpressing the *B. subtilis goxB* gene was ~ 4.E-03 U/mg proteins (corresponding to ~ 0.004 U/mL), against 0.4 U/mL reported in literature for the purified enzyme [28].

The effect of glycine oxidase overexpression on butanol and isobutanol accumulation was preliminary tested in the two yeast genetic backgrounds, grown like described for Figure 2. In both cases, in the strains overexpressing the *goxB* opt gene we observed a higher butanol and isobutanol accumulation, being about 30% and 15%, respectively (Figure 3, dark and pale gray columns, respectively). The data prove that an activity responsible for glycine conversion into glyoxylate is the first step leading to butanol accumulation, and we also show a first example of how to improve said activity (Figure 3, upper bars).

### The second reaction of the pathway: from glyoxylate to β-ethylmalate

**Figure 4:**
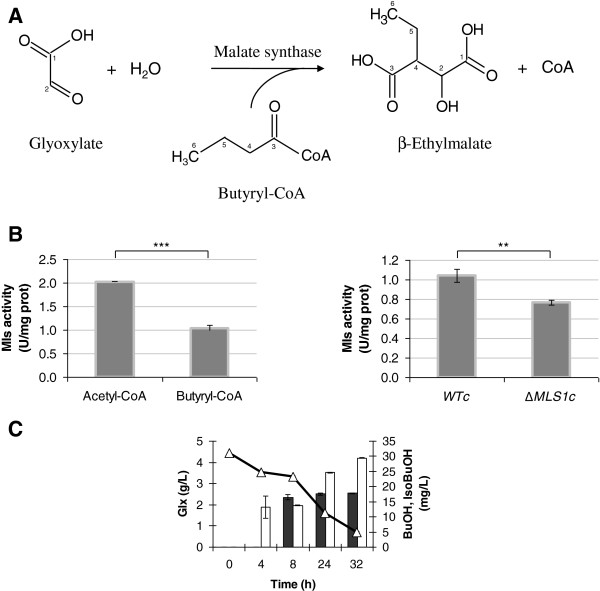
**Malate synthase activity and glyoxylate bioconversion into butanol and isobutanol.** (**A**) Glyoxylate conversion reaction performed by malate synthase enzyme in the presence of butyryl-CoA. The carbons of the molecules were numbered. (**B**) Malate synthase activity for the glyoxylate conversion into β-ethylmalate. Left panel: malate synthase activity was assayed using acetyl-CoA or butyryl-CoA as donor group. Right panel: effect of *MLS*1 deletion on the enzymatic activity. The data presented here are representative of three independent experiments. p ≤ 0,05 = *; p ≤ 0,01 = **; p ≤ 0,001 = ***; p > 0,05 = n.s. (**C**) Glyoxylate bioconversion. Butanol (full columns) and isobutanol (empty columns) production as well as glyoxylate consumption (triangle) measured at different time point. The data presented here are representative of three independent experiments.

In yeasts, no activity able to catalyze the condensation of glyoxylate with butyryl-CoA to form β-ethylmalate has been described yet. However, the malate synthase enzyme catalyzes a similar reaction: the glyoxylate condensation with acetyl-CoA to form malate [29]. In *S. cerevisiae* two isoforms of malate synthase are described, encoded by *MLS*1 and *DAL*7 genes [29]. *MLS*1 expression is repressed by glucose and induced when cells are growing on non-fermentable carbon sources, such as fatty acid, ethanol or acetate. *DAL*7 encodes for an enzyme recycling the glyoxylate generated during allantoin degradation. Its expression is controlled by the nitrogen source present in the medium, resulting repressed in the presence of rich nitrogen sources, such as asparagine and ammonium, and derepressed in the presence of poor nitrogen sources, such as proline [29]. We hypothesized first and then evaluated if butyryl-CoA could also be a substrate of malate synthase enzyme, considering the structural analogy with acetyl-CoA, Figure 4A.

Yeasts were grown in rich YPD medium and malate synthase activity was detected in the presence of acetyl-CoA or butyryl-CoA as acyl-CoA donors, using a modified TNB-based assay (see Methods) (Figure 4B, left panel). YPD medium was used to have a cultural condition in which both Mls1 and Dal7 were present at similar level, as reported in literature [29]. Remarkably, when butyryl-CoA was added as acyl-CoA donor a malate synthase activity was detected, even if at lower value if compared to the activity measured in the presence of acetyl-CoA (1 U/mg proteins *versus* 2 U/mg proteins, respectively). Based on our information, this is the first experimental evidence that a yeast malate synthase can accept butyryl-CoA as acyl-CoA donor. The *MLS*1 deletion negatively affects the activity (about 25% of reduction, Figure 4B, right panel) and the same impairment is caused by *DAL*7 deletion (data not shown), suggesting that the two enzymes might similarly contribute to the reaction of interest.

If our hypothesis is correct, feeding the cells with glyoxylate should result in butanol and isobutanol accumulation. To determine favourable production conditions, yeast cells were grown in minimal medium in the absence or presence of different amount of glyoxylate (0.5, 1, 5 g/L) at different pH (2.5 and 5.5) values. Figure 4C confirms the butanol and isobutanol accumulation, not detectable if glyoxylate is absent (data not shown). In the reported example the production was obtained starting with 5 g/L of glyoxylate at pH 2.5. This pH value has been selected to facilitate the diffusion of undissociated glyoxylic acid inside the cells, since no carrier for this metabolite is reported in literature. In this condition the glyoxylate was almost totally consumed. Cells accumulated during the time about 20 and 30 mg/L of butanol and isobutanol, respectively. This confirms the involvement of glyoxylate as intermediate in the formation of the desired alcohols. At the moment we have no explanation for the higher isobutanol accumulation. One possibility could be that in case of high amount of glyoxylate, Agx1 [30] catalyzes its conversion into glycine, shifting the reaction trough serine formation (see Figure 1).

### The third reaction of the pathway: from β-ethylmalate to α-ketovalerate

**Figure 5:**
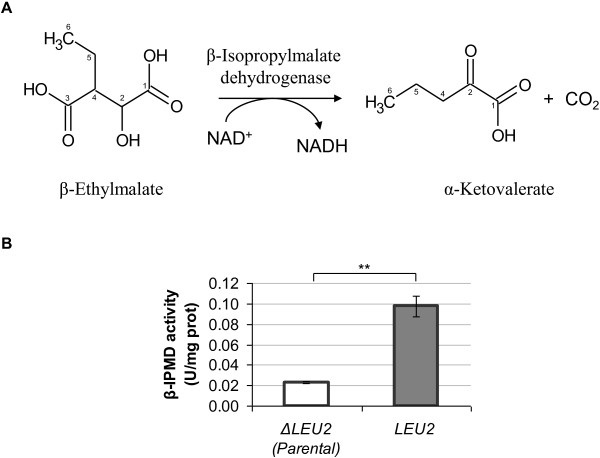
**The β-isopropylmalate dehydrogenase involvement in the glycine degradation pathway.** The β-isopropylmalate dehydrogenase activity was tested in a coupled reaction using glyoxylate as substrate instead of β-ethylmalate (not commercial available). The carbons of the molecules were numbered. (**A**) Reaction catalysed by the β-isopropylmalate dehydrogenase enzyme. (**B**) Activity of β-isopropylmalate dehydrogenase measured in yeast strain Δ*LEU*2 (empty column) and overexpressing *LEU*2 gene (grey column). The data presented here are representative of three independent experiments. p ≤ 0,05 = *; p ≤ 0,01 = **; p ≤ 0,001 = ***; p > 0,05 = n.s.

In *E. coli* the β-isopropylmalate dehydrogenase enzyme, encoded by the *leuB* gene, catalyzes the conversion of β-isopropylmalate into α-ketoisocaproate. Shen and Liao [22] have demonstrated the possibility to use this enzyme to additionally catalyze the conversion of β-ethylmalate into α-ketovalerate in *E. coli* cells (the reaction is depicted in Figure 5A). *LEU*2 is the homologous of *leuB* in *S. cerevisiae*[31]. Therefore, by using the BY4741 strain (deleted in *LEU*2) and the same strain transformed with a *LEU*2 multicopy plasmid, we tested the contribution of the Leu2 activity in the novel pathway. The activity assay was performed using two coupled reactions, since β-ethylmalate is not commercially available. In the first reaction, glyoxylate and butyryl-CoA should be converted into β-ethylmalate by the malate synthase activity, as described in the previous paragraph. The second coupled reaction, catalyzed by the Leu2 activity, utilizes the produced β-ethylmalate to generate α-ketovalerate and releasing NADH as reduced equivalent. Said cofactor can be spectrophotometrically measured to determine the activity. In the *LEU*2 overexpressing strain the activity was about 5 fold higher when compared to the Δ*LEU*2 strain, being 0.1 and 0.02 U/mg proteins, respectively (Figure 5B). When *MLS*1 or *DAL*7 are deleted while Leu2 is present, the dehydrogenase activity significantly decreases, confirming that β-ethylmalate is the substrate of the reaction (Table 1). Table 1 reports all the combinations tested and the registered activities. Interestingly, the residual activity detected whenever *LEU*2 is deleted suggests that other(s) activity(ies) might be responsible for β-ethylmalate conversion.

**Table 1:** β-Isopropylmalate dehydrogenase activity measured with assay coupling the reactions catalyzed by Mls1 and Leu2

**Gene**			**β-IPMD Activity (U/mg prot)**
***MLS1***	***DAL7***	***LEU2***	
+	+	++	0.098 ± 0.010
+	+	-	0.023 ± 0.001
+	-	++	0.019 ± 0.002
+	-	-	0.016 ± 0.002
-	+	++	0.021 ± 0.002
-	+	-	0.018 ± 0.002

The symbols “+” indicate the wild type copy or “++” the overexpression of the corresponding gene in the table. The symbol “-” indicate the deletion of the gene. The data presented here are representative of three independent experiments.

Concluding, these data indicate that the presence of *LEU*2 coupled with malate synthase activity guarantees the glyoxylate conversion into α-ketovalerate.

### The last step: from α-ketoacids to butanol and isobutanol

**Figure 6:**
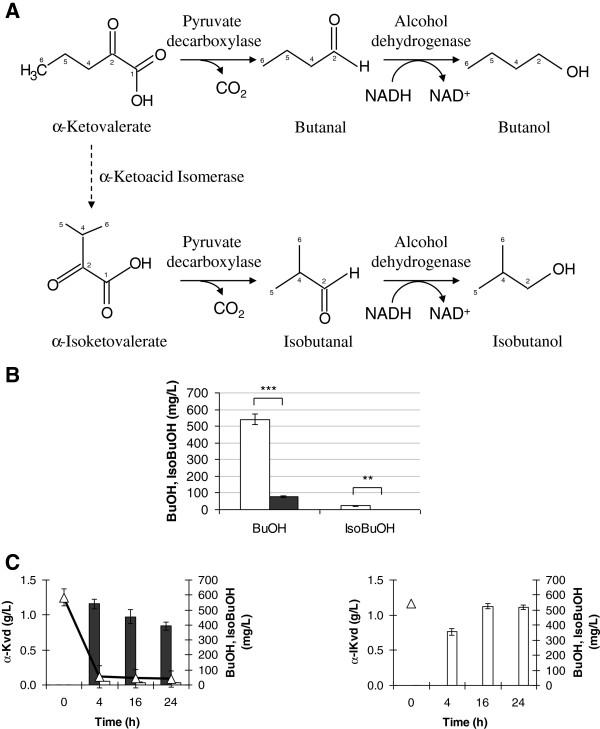
**Pyruvate decarboxylase activity and α-ketoacids bioconversion into butanol and isobutanol.** (**A**) α-ketovalerate and α-isoketovalerate conversion reaction performed by pyruvate decarboxylase enzyme. The carbons of the molecules were numbered. (**B**) Pyruvate decarboxylase deletion effect on butanol and isobutanol accumulation using α-ketovalerate as substrate. Wild type (empty column) and *PCD*1, 5, 6 deleted strains (full column). The data presented here are representative of three independent experiments. p ≤ 0,05 = *; p ≤ 0,01 = **; p ≤ 0,001 = ***; p > 0,05 = n.s. (**C**) α-Ketovalerate (left panel) and α-isoketovalerate (right panel) bioconversion. Butanol (dark grey), isobutanol (white) and α-ketovalerate consumption (triangle) were reported. For α-isoketovalerate (triangle), the sole estimated initial concentration is given. The data presented here are representative of three independent experiments.

The last step necessary in the glycine degradation to produce the desired alcohols is the reductive decarboxylation of α-ketovalerate into butanol and of α-isoketovalerate into isobutanol, Figure 6A.

The conversion of α-ketovalerate into α-isoketovalerate was proposed to occur through a dehydratation reaction, catalyzed by dihydroxyacid dehydratase enzyme [21]. However, at the best of our information, no experimental evidences are reported up to now. By looking at the chemical structure of the two ketoacids, this reaction might require an isomerization, like proposed in Figure 1.

The conversion of α-ketovalerate into butanol requires two reactions: in the first one α-ketovalerate decarboxylation generates the corresponding aldehyde; in the second one the aldehyde is reduced to alcohol, butanol in this case (Figure 6A, upper part). It was reported that in *S. cerevisiae* α-keto-β-methylvalerate, α-ketoisocaproate and α-isoketovalerate [32] can be decarboxylated to the α-ketoacids by pyruvate decarboxylases (Pdc). Going one step forward, Brat *et al.* reported that through the α-isoketovalerate decarboxylation isobutanol can be produced [16], starting from valine as substrate. However, to avoid co-current pyruvate decarboxylation into ethanol, the authors replaced the *PDC* genes with the decarboxylase encoded by *ARO*10 gene, which has no activity on pyruvate [16].

Yeast no data are available about a possible α-ketovalerate decarboxylation in yeast. When pyruvate, α-ketovalerate and α-isoketovalerate were used as substrates, the activities measured using an assay for Pdc activity were 722 ± 2.3, 1.75 ± 0.3 and 0.3 ± 0.05 U/mg proteins, respectively. The involvement of pyruvate decarboxylase in the last reaction was also demonstrated by measuring the butanol and isobutanol production in a *PDC*1, 5, 6 deleted strain. In the presence of α-ketovalerate (1.1612 g/L, 10 mM) as substrate, the deletion of all three isoforms of pyruvate decarboxylase significantly decreases the butanol and isobutanol production, as shown in bioconversion experiment (Figure 6B). In particular, the butanol titer was 5 times lower than in wild type strain, 118 *versus* 583 mg/L respectively. By incubating the triple *PDC* deleted strain with α-isoketovalerate no isobutanol accumulation was observed (data not shown).

It is important to underline that in the presence of α-ketovalerate as substrate both butanol and isobutanol are produced (Figure 6C, left panel) in wild type strain. Considering that more isobutanol is obtained when glycine is added to glucose minimal medium (Figure 2), we could hypothesize that α-isoketovalerate might partially derive from α-ketovalerate and that this reaction is probably irreversible, since in the presence of α-isoketovalerate only isobutanol accumulation was observed (Figure 6C, right panel).

### The glyoxylate conversion into butanol and isobutanol requires Mls1, Leu2 and Pdc(s) activities

To further prove the link between glycine and the fusel alcohols we developed an *in vitro* assay in which all reactions of the proposed pathway are coupled. To perform this assay we measured the pyruvate decarboxylase activity using glycine as substrate, monitoring the decrement of OD_340nm_ since NADH is consumed during the last reaction. No data were obtained. We believe that this could be related to (*i*) a lower conversion of glycine to glyoxylate and/or to (*ii*) the very different physio-chemical assay conditions required by glycine oxidase (the first enzyme of the pathway) in respect to all the other enzymes. Indeed, a Pdc activity was detected when glyoxylate was used as substrate, confirming the conversion of glyoxylate to the α-ketovalerate.

**Figure 7:**
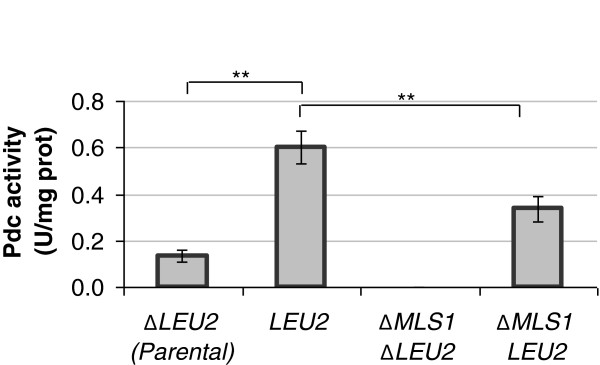
**The evidence of glyoxylate degradation through the Mls, β-IPMD and Pdc(s) activities.** The *MLS*1 deletion effect was evaluated coupled with the *LEU*2 deletion or overexpression (respectively indicated as Δ*MLS*1*ΔLEU*2 and Δ*MLS*1 *LEU*2). The data presented here are representative of three independent experiments. p ≤ 0,05 = *; p ≤ 0,01 = **; p ≤ 0,001 = ***; p > 0,05 = n.s.

Figure 7 shows the complete panel of the Pdc activities measured in the different strains. The higher activity was registered in the wild type strain overxpressing the *LEU*2 gene, the minimal (almost undetectable) in the double *MLS*1/*LEU*2 deleted strain and an intermediate situation when only one of the two genes was deleted. To be more precise, the *LEU*2 deletion affects more the activity, very likely because in the case of *MLS*1 deletion, Dal7 is able to replace its function (and vice versa) [29].

## Conclusions

This study describes a novel pathway to produce butanol and isobutanol in the yeast *S. cerevisiae* through the glycine degradation pathway.

We characterized the entire pathway identifying for each step at least one enzymatic reaction with at least one relative gene for butanol production. 92 mg/L of butanol were produced starting from glycine as substrate.

It might be speculated that butanol derives from butyryl-CoA and that glycine is solely used to drive the flux. Remarkably, the proposed pathway implies catalytic reactions that justify how glycine is not simply burnt to carbon dioxide, but is an effective co-substrate for the butanol production, as highlighted by the numbered carbons (see Figures 3, 4, 5 and 6, panels A).

Despite the yield on glycine is still quite low, it should be underlined that butanol was obtained through endogenous activities which are in general involved in other reactions and specific for other substrates. Therefore, it can be anticipated that there are many different possibilities for optimizing the pathway, considering every single enzyme involved, the pool of substrates and their compartmentalization.

From an economical point of view the production of higher alcohols starting from purified glycine cannot be considered as a sustainable process. Metabolic engineering and synthetic biology can then help in the construction of a yeast redirecting sugars to glycine production, and from there to optimize the sole butanol production, but this appears at the moment as a long way to run. However, an alternative seems closer to reality: a considerable fraction of proteins accumulate as waste product deriving from exhausted biomasses of different microbial productions and currently is not fully absorbed by the market [12]. It is then possible to imagine a side-stream process of production which is based on protein hydrolysates; the different aminoacids fed to a yeast strain optimized for desired fusel alcohol production could generate a blend with good properties as biofuel [12], adding value and completing the concept of biorefinery.

Considering the potentiality of *S. cerevisiae* as ethanol producer and considering the potentiality of the aminoacid degradation pathway for fusel alcohol productions [12], it really seems the right moment to intensify the effort in studying and improving yeast tolerance to a mixture of different organic solvents.

## Methods

### Strains and growth conditions

The *S. cerevisiae* strains used in this study were CEN.PK 102-5B (*MATa,ura3-52, his3-11, leu2-3/112, TRP1, MAL2-8c, SUC2* - Dr. P. Kötter, Institute of Microbiology, Johann Wolfgang Goethe-University, Frankfurt, Germany) [33] and BY4741 (*MATa, ura3Δ0, leu2Δ0, met15Δ0, his 3Δ1*) (EUROSCARF collection, Heidelberg, Germany). The strains BY4741Δ*MLS*1 (*MATa; his3 Δ1; leu2 Δ0; met15 Δ0; ura3 Δ0; YNL117w::kanMX4*) and BY4741Δ*DAL*7 (*MATa; his3 Δ1; leu2 Δ0; met15 Δ0; ura3 Δ0; YIR031c::kanMX4*) are provided by EUROSCARF deleted strain collection (EUROSCARF collection, Heidelberg, Germany). The strain deleted in all three isoform of pyruvate decarboxylase (*ΔPDC*1, 5, 6) is CEN.PK RWB837 (*MATa; pdc1::loxP, pdc5::loxP, pdc6::loxP, ura3-52*) [34]. Strains designed with “c” correspond to the respective parental strain transformed with empty plasmid(s) (see below) to render them prototrophic. Strains designed with “*goxB* opt” are the corresponding parental strain transformed with plasmid pYX212 (see below) with the *B. subtilis goxB* coding sequence optimized for the *S. cerevisiae* codon usage. *goxB* opt gene was expressed under the control of the *S. cerevisiae TPI*1 promoter. Yeast transformations were performed according to the LiAc/PEG/ss-DNA protocol [35]. All the parental and transformed strains are reported in the Table 2.

**Table 2:** Strains and plasmids used in this work

**Parental strain**		
**Strain**	**Genotype**	
CEN.PK 102-5B	*MATa, ura3-52, his3-11, leu2-3/112, TRP1, MAL2-8c, SUC2*	
CEN.PK RWB837	*MATa, pdc1::loxP, pdc5::loxP, pdc6::loxP, ura3-52*	
BY4741	*MATa, ura3*Δ*0,leu2*Δ*0*, *met15*Δ*0,his 3*Δ*1*	
BY4741Δ*MLS*1	*MATa; his3* Δ*1*; *leu2* Δ*0; met15* Δ*0; ura3* Δ*0; YNL117w::kanMX4*	
BY4741Δ*DAL*7	*MATa; his3 Δ1; leu2 Δ0; met15 Δ0; ura3 Δ0;YIR031c::kanMX4*	
**Plasmids**		
**Expression plasmid**	**Segregation**	**Markers, genes**
pYX212	multicopy (2μ derived)	*URA*3
pYX212*goxB* opt	multicopy (2μ derived)	*URA*3, optimised *goxB*
pYX022	integrative	*HIS*3
pYX242	multicopy (2μ derived)	*LEU*2
**Transformed strain**		
**Strain**	**Plasmid**	**Obtained strain**
CEN.PK 102-5B	pYX212, pYX022, pYX242	CEN.PKc
BY4741	pYX212, pYX022, pYX242	BY4741c
BY4741	pYX212*goxB* opt, pYX022, pYX242	BY4741c *goxB* opt
BY4741Δ*MLS*1	pYX212, pYX022, pYX242	BY4741ΔMLS1c
BY4741Δ*MLS*1	pYX212*goxB* opt, pYX022, pYX242	BY4741ΔMLS1c *goxB* opt
BY4741Δ*DAL*7	pYX212, pYX022, pYX242	BY4741ΔDAL7c

### Media composition for cell growth and bioconversion

Independent transformants and the respective control strains (at least three for each transformation) were cultivated in shake flasks with 5/1 ratio of flask volume/medium in minimal synthetic medium with 20 g/L of glucose and supplemented with glycine, glyoxylate, α-ketovalerate or α-isoketovalerate, as specifically indicated in the experiments. YPD and YPGE (for the triple *PDC* deleted strains) media were prepared as follows: yeast extract 1% (w/v), tryptone 2% (w/v) and glucose 2% (w/v) for the YPD. In the YPGE the glucose was replaced with glycerol 1% (v/v) and ethanol 1% (v/v). All strains were grown at 30°C on orbital shaker at 160 rpm for 72 hours.

#### Kinetic experiment

The butanol and isobutanol production starting from glycine (Figure 2) was performed by growing the cells in Verduyn medium [36] with glucose 20 g/L and glycine 15 g/L as substrates.

#### Bioconversion experiment

The bioconversion experiments were performed in two phases: 1) cells were grown in YPD or YPGE (for the triple *PDC* deleted strains) medium until the stationary phase; 2) cells were collected by centrifugation (10 min at 4000 rpm) washed once with water and inoculated in appropriate medium to perform the bioconversion phase. The medium for glyoxylate bioconversion (Figure 4C) was minimal synthetic medium with glucose 20 g/L and glyoxylate 5 g/L at pH 2.5. The medium for α-ketovalerate (or α-isoketovalerate) bioconversion (Figure 6C) was minimal synthetic medium with glucose 20 g/L and α-ketovalerate (or α-isoketovalerate) 1.1612 g/L (corresponding to 10 mM).

### Gene amplification and plasmids construction

The *B. subtilis goxB* gene was designed with codon usage adaptation for *S. cerevisiae* by Eurofins MWG Operon. In the Additional file 1 was reported the complete sequence of *goxB* synthesized. *goxB* opt gene was subcloned into the multicopy yeast expression plasmid pYX212 (R&D Systems, Inc., Wiesbaden, D, *URA*3 marker), resulting in the plasmid pYX212*goxB* opt. The heterologous gene is under the control of the *S. cerevisiae TPI*1 promoter. For the construction of the plasmid pYX212*goxB* opt, the recipient vector was *EcoR*I cut, blunted and dephosphorylated, while the insert was *EcoR*I blunt excised from the Eurofins plasmid. DNA manipulation, transformation and cultivation of *E. coli* (Novablue, Novagen) were performed following standard protocols [37]. All the restriction and modification enzymes utilised are from NEB (New England Biolabs, UK) or from Roche Diagnostics.

### Cell growth and metabolites determination

The cellular growth was spectrophotometrically monitored at 660nm and was reported as variation of the optical density (OD) as a function of time (h). The amount of extracellular glucose, butanol, isobutanol, glyoxylate and α-ketovalerate were determined by HPLC based method using H_2_SO_4_ 5 mM as mobile phase and Aminex HPX-87P column, 300 × 7.8 mm with a polystyrene divinylbenzene-based matrix (BioRad). The glycine quantification was performed using a previously described assay [38].

### Determination of enzymatic activities

Exponentially growing cells were harvested by centrifugation at 4000 rpm for 10 min and washed with cold deionised water. The cell pellet was then re-suspended in 25 mM Tris-HCl pH 8.0 with protease inhibitor cocktail (Roche diagnostics, Cat. No. 04906837001) and 1 mM of phenylmethylsulfonyl fluoride (PMSF) and mechanically disrupted using glass microbeads (600 μm, Sigma-Aldrich). Cells debris was removed by centrifugation at 14000 rpm for 10 min at 4°C and the clarified crude extract was used for enzymatic analysis. The protein concentration in cell-free extracts was estimated according to Bradford [39] using bovine serum albumin as reference.

Enzyme activities were measured on cell-free extracts by spectrophotometric assays. Activities were expressed as U/mg of total proteins.

#### Glycine oxidase activity

Glycine oxidase activity was assayed spectrophotometrically via determination of H_2_O_2_ with an enzyme-coupled assay using horseradish peroxidase and *o*-dianisidine, as previously described with some modifications [28]. The assay was performed on a final volume of 1 ml as follows: Tris-HCl 100 mM pH 8, phosphoric acid 10 mM, glycine 50 mM, *o*-dianisidine 1 mM, FAD 0.198 μM, horseradish peroxidase 14.72 U/mL, cell-free protein extract (0.5 mg/mL). The reaction was incubated at 37°C for 90 minutes and yellow colour, developed by o-dianisidine oxidation, was monitored at 530 nm. The glycine oxidase activity was expressed as U/mg of total proteins using the following equation:

$$\begin{array}{c} \mathrm{Activity}\;\left(U/\mathrm{mg}\;\mathrm{prot}\;\mathrm{tot}\right)\\ \;=\left(\left(\left(\mathrm{OD}\;530\mathrm{nm}/\min\right)/\epsilon \right)\cdot \cdots \mathrm{dilution}\;\mathrm{factor}\right)/\mathrm{mg}\;\mathrm{prot}\;\mathrm{tot} \end{array}$$

Were ϵ = 8.3 · 1/(mM · cm)

One glycine oxidase unit is defined as the amount of enzyme that converts 1 mole of substrate (glycine) per minute at 25°C.

#### Malate synthase activity

The malate synthase activity was performed as described in Sigma-Aldrich protocol [40] using acetyl-CoA (or butyryl-CoA) + glyoxylate. The assay take into consideration that glyoxylate condensation with acetyl-CoA (or butyryl-CoA) produces malate (or β-ethylmalate) and CoA. The free CoA can react with the Ellman reagent DTNB (5,5'-Dithio-bis(2-Nitrobenzoic Acid)) which reacts with free thiol groups, producing CoA-derivative and TNB (5-Thio-2-Nitrobenzoic Acid) [41]. The quantity of TNB produced is in stoichiometric ratio (1:1) with free thiol groups and was monitored spectrophotometrically at 412 nm.

#### β-isopropylmalate dehydrogenase activity (using glyoxylate and butyryl-CoA as substrates)

The β-isopropylmalate dehydrogenase enzyme catalyzes the NAD-dependent oxidation of the substrate with simultaneously conversion of NAD^+^ to NADH. The activity was spectrophotometrically determined at 340 nm. The assay was performed on a final volume of 1 mL in cuvette with imidazole 50 mM pH 8, MgCl_2_ 10 mM, butyryl-CoA 0.125 mM, glyoxylate 0.5 mM, NAD^+^ 1.575 mM. After incubation at 30°C for 10 min cell-free protein extract (0.6 mg/mL) were added and increasing of absorbance at 340 nm was monitored for 10 min.

The β-isopropylmalate dehydrogenase activity was expressed as U/mg total proteins using the following equation:

$$\begin{array}{c} \mathrm{Activity}\;\left(U/\mathrm{mg}\;\mathrm{prot}\;\mathrm{tot}\right)\\ \;=\left(\mathrm{OD}\;340\mathrm{nm}/\min\cdot \cdots \mathrm{dilution}\;\mathrm{factor}\right)/\epsilon \cdot \cdots \mathrm{Ev} \end{array}$$

Were ϵ is the millimolar extinction coefficient of NADH at 340 nm (6.22 · 1/(mM · cm)) and Ev is the volume of cell extract used (expressed in millilitres).

#### Pyruvate decarboxylase activity (using glycine or glyoxylate and butyryl-CoA as substrates)

The pyruvate decarboxylase enzyme catalyzes the decarboxylation of ketoacid to form the derived aldehyde which is reduced by alcohol-dehydrogenase NADH-dependent activity. The conversion of NADH to NAD^+^ is spectrophotometrically revealed at 340 nm.

The assay was performed based on pyruvate decarboxylase assay protocol of Sigma-Aldrich [40] with some modifications. When glycine was used as substrate Tris-HCl 100 mM pH 8, phosphoric acid 10 mM, glycine 50 mM, FAD 0.198 μM, MgCl_2_ 10 mM, butyryl-CoA 0.125 mM and NADH 0.16 mM were added in cuvette in a final volume of 1 mL. After incubation at 37°C for 30 minutes, alcohol dehydrogenase enzymatic solution (200 U/mL) and 0.2 mg/mL of cell-free extract were added. The decrease of absorbance at 340 nm was monitored for 15–30 min.

When glyoxylate and butyryl-CoA were used as substrates imidazole buffer 35 mM, MgCl_2_ 10 mM, butyryl-CoA 0.125 mM, glyoxylate 0.5 mM and NADH 0.16 mM were added in cuvette in a final volume of 1 mL. After incubation at 30°C for 10 min 20 μL of alcohol dehydrogenase enzyme solution (200 U/mL) and 0.2 mg/mL of cell-free extract were added. The decrease of absorbance at 340 nm was monitored for 15 min.

The activity was expressed as U/mg total proteins using the following equation: Activity (U/mg prot tot) = (OD 340nm/min · dilution factor)/ϵ · Ev.

Were ϵ is the millimolar extinction coefficient of NADH at 340 nm (6.22 · 1/(mM · cm)) and Ev is the volume of cell extract used (expressed in millilitres).

## Abbreviations

GAP1: General Amino acid Permease gene; Shm2: Serine HydroxyMethyltransferase enzyme; GDC: Glycine Decarboxylase (enzymatic) Complex; goxB: Glycine OXidase gene, *B. subtilis*; MLS1: MaLate Synthase gene; DAL7: Malate synthase gene (name description: Degradation of ALlantoin); Agx1: Alanine:Glyoxylate aminotrans(X)ferase enzyme; leuB: LEUcine Biosynthesis (β-isopropylmalate dehydrogenase gene), *E. coli*; LEU2: LEUcine biosynthesis (β-isopropylmalate dehydrogenase gene); β-IPMD: β-IsopropylMalate Dehydrogenase enzyme; pTPI: Triose-Phosphate Isomerase, promoter; PDC: Pyruvate DeCarboxylase gene(s); ARO10: AROmatic amino acid requiring gene; NAD(H)+: Nicotinamide Adenine Dinucleotide (oxidized and reduced); FAD(H2): Flavin Adenine Dinucleotide (oxidized and reduced); -CoA: Coenzyme A; YPD: Yeast extract, Peptone, Dextrose; YPGE: Yeast extract, Peptone, Glycerol, Ethanol; PMSF: PhenylMethaneSulfonyl Fluoride; DTNB/TNB: 5,5'-DiThio-bis(2-Nitrobenzoic Acid)/5-Thio-2-Nitrobenzoic Acid; OD: Optical Density; HPLC: High-Performance Liquid Chromatography; *: if not differently specified, the listed genes and enzymes refer to *S. cerevisiae*

## Competing interests

PB recently applied as inventor of a patent application related to the content of the manuscript. In any way PB, or whatever organization, will gain or lose financially from the publication of this manuscript, either now nor in the future. The other authors declare that they have no competing interests.

## Authors’ contributions

VL carried out the experiments for butanol and isobutanol production and the enzymatic assays, participated in the evaluation of the data and in compiling the manuscript. NB developed the *goxB* optimized strains and took part in the data analysis. GR carried out the bioconversion experiments on alpha-ketoacids and took part in the data analysis. DP participated in the experimental work design and contributed to the data interpretation. PB conceived the study, participated in its design and compiled the manuscript. All the authors have read and approved the final manuscript.

## Supplementary Material

Additional file 1 **Sequence of synthesized *goxB *gene with codon usage optimized for *Saccharomyces cerevisiae.*** Click here for file

## References

[B1] Savage N Fuel options: The ideal biofuel Nature 2011 474 S9 S11 21697843 10.1038/474S09a

[B2] Dong H Tao W Dai Z Yang L Gong F Zhang Y Li Y Biobutanol Adv Biochem Eng Biotechnol 2012 128 85 100 22167047 10.1007/10_2011_128

[B3] Atsumi S Cann AF Connor MR Shen CR Smith KM Brynildsen MP Chou KJ Hanai T Liao JC Metabolic engineering of *Escherichia coli* for 1-butanol production Metab Eng 2008 10 305 311 10.1016/j.ymben.2007.08.003 17942358

[B4] Steen EJ Chan R Prasad N Myers S Petzold CJ Redding A Ouellet M Keasling JD Metabolic engineering of *Saccharomyces cerevisiae* for the production of n-butanol Microb Cell Fact 2008 7 36 10.1186/1475-2859-7-36 19055772 PMC2621116

[B5] Bond-Watts BB Bellerose RJ Chang MC Enzyme mechanism as a kinetic control element for designing synthetic biofuel pathways Nat Chem Biol 2011 7 222 227 10.1038/nchembio.537 21358636

[B6] Trinh CT Elucidating and reprogramming *Escherichia coli* metabolisms for obligate anaerobic n-butanol and isobutanol production Appl Microbiol Biotechnol 2012 95 1083 1094 10.1007/s00253-012-4197-7 22678028

[B7] McKee AE Rutherford BJ Chivian DC Baidoo EK Juminaga D Kuo D Benke PI Dietrich JA Ma SM Arkin AP Manipulation of the carbon storage regulator system for metabolite remodeling and biofuel production in *Escherichia coli* Microb Cell Fact 2012 11 79 10.1186/1475-2859-11-79 22694848 PMC3460784

[B8] Ohno S Furusawa C Shimizu H In silico screening of triple reaction knockout *Escherichia coli* strains for overproduction of useful metabolites J Biosci Bioeng 2013 115 221 228 10.1016/j.jbiosc.2012.09.004 23041138

[B9] Shen CR Lan EI Dekishima Y Baez A Cho KM Liao JC Driving forces enable high-titer anaerobic 1-butanol synthesis in *Escherichia coli* Appl Environ Microbiol 2011 77 2905 2915 10.1128/AEM.03034-10 21398484 PMC3126405

[B10] Lan EI Liao JC **Microbial synthesis of n-butanol, isobutanol, and other higher alcohols from diverse resources** Bioresour Technol 2013 135 339 349 23186690 10.1016/j.biortech.2012.09.104

[B11] Dellomonaco C Clomburg JM Miller EN Gonzalez R Engineered reversal of the β-oxidation cycle for the synthesis of fuels and chemicals Nature 2011 476 355 359 10.1038/nature10333 21832992

[B12] Huo YX Cho KM Rivera JG Monte E Shen CR Yan Y Liao JC Conversion of proteins into biofuels by engineering nitrogen flux Nat Biotechnol 2011 29 346 351 10.1038/nbt.1789 21378968

[B13] Chen X Nielsen KF Borodina I Kielland-Brandt MC Karhumaa K Increased isobutanol production in *Saccharomyces cerevisiae* by overexpression of genes in valine metabolism Biotechnol Biofuels 2011 4 21 10.1186/1754-6834-4-21 21798060 PMC3162486

[B14] Kondo T Tezuka H Ishii J Matsuda F Ogino C Kondo A Genetic engineering to enhance the Ehrlich pathway and alter carbon flux for increased isobutanol production from glucose by *Saccharomyces cerevisiae* J Biotechnol 2012 159 32 37 10.1016/j.jbiotec.2012.01.022 22342368

[B15] Lee WH Seo SO Bae YH Nan H Jin YS Seo JH Isobutanol production in engineered *Saccharomyces cerevisiae* by overexpression of 2-ketoisovalerate decarboxylase and valine biosynthetic enzymes Bioprocess Biosyst Eng 2012 35 1467 1475 10.1007/s00449-012-0736-y 22543927

[B16] Brat D Weber C Lorenzen W Bode HB Boles E Cytosolic re-localization and optimization of valine synthesis and catabolism enables inseased isobutanol production with the yeast *Saccharomyces cerevisiae* Biotechnol Biofuels 2012 5 65 10.1186/1754-6834-5-65 22954227 PMC3476451

[B17] Brat D Boles E Isobutanol production from d-xylose by recombinant *Saccharomyces cerevisiae* FEMS Yeast Res 2013 13 241 244 10.1111/1567-1364.12028 23279585

[B18] Hofman-Bang J Nitrogen catabolite repression in *Saccharomyces cerevisiae* Mol Biotechnol 1999 12 35 73 10.1385/MB:12:1:35 10554772

[B19] McNeil JB McIntosh EM Taylor BV Zhang FR Tang S Bognar AL Cloning and molecular characterization of three genes, including two genes encoding serine hydroxymethyltransferases, whose inactivation is required to render yeast auxotrophic for glycine J Biol Chem 1994 269 9155 9165 8132653

[B20] Sinclair DA Dawes IW Genetics of the synthesis of serine from glycine and the utilization of glycine as sole nitrogen source by *Saccharomyces cerevisiae* Genetics 1995 140 1213 1222 7498764 10.1093/genetics/140.4.1213 PMC1206688

[B21] Villas-Bôas SG Kesson M Nielsen J Biosynthesis of glyoxylate from glycine in *Saccharomyces cerevisiae* FEMS Yeast Res 2005 5 703 709 10.1016/j.femsyr.2005.03.001 15851099

[B22] Shen CR Liao JC Metabolic engineering of *Escherichia coli* for 1-butanol and 1-propanol production via the keto-acid pathways Metab Eng 2008 10 312 320 10.1016/j.ymben.2008.08.001 18775501

[B23] Atsumi S Hanai T Liao JC Non-fermentative pathways for synthesis of branched-chain higher alcohols as biofuels Nature 2008 451 86 89 10.1038/nature06450 18172501

[B24] Nishiya Y Imanaka T Purification and characterization of a novel glycine oxidase from *Bacillus subtilis* FEBS Lett 1998 438 263 266 10.1016/S0014-5793(98)01313-1 9827558

[B25] Rabin R Reeves HC Ajl SJ Beta-ethylmalate synthetase J Bacteriol 1963 86 937 944 14080804 10.1128/jb.86.5.937-944.1963 PMC278549

[B26] Hazelwood LA Daran JM van Maris AJ Pronk JT Dickinson JR The Ehrlich pathway for fusel alcohol production: a century of research on *Saccharomyces cerevisiae* metabolism Appl Environ Microbiol 2008 74 2259 2266 10.1128/AEM.02625-07 18281432 PMC2293160

[B27] Piper MD Hong SP Eissing T Sealey P Dawes IW Regulation of the yeast glycine cleavage genes is responsive to the availability of multiple nutrients FEMS Yeast Res 2002 2 59 71 12702322 10.1111/j.1567-1364.2002.tb00069.x

[B28] Job V Marcone GL Pilone MS Pollegioni L Glycine oxidase from *Bacillus subtilis*. Characterization of a new flavoprotein J Biol Chem 2002 277 6985 6993 10.1074/jbc.M111095200 11744710

[B29] Hartig A Simon MM Schuster T Daugherty JR Yoo HS Cooper TG Differentially regulated malate synthase genes participate in carbon and nitrogen metabolism of *S. cerevisiae* Nucleic Acids Res 1992 20 5677 5686 10.1093/nar/20.21.5677 1454530 PMC334402

[B30] Schlösser T Gätgens C Weber U Stahmann KP Alanine : glyoxylate aminotransferase of *Saccharomyces cerevisiae*-encoding gene *AGX*1 and metabolic significance Yeast 2004 21 63 73 10.1002/yea.1058 14745783

[B31] Kohlhaw GB Beta-isopropylmalate dehydrogenase from yeast Methods Enzymol 1988 166 429 435 3071718 10.1016/s0076-6879(88)66056-3

[B32] ter Schure EG Flikweert MT van Dijken JP Pronk JT Verrips CT Pyruvate decarboxylase catalyzes decarboxylation of branched-chain 2-oxo acids but is not essential for fusel alcohol production by *Saccharomyces cerevisiae* Appl Environ Microbiol 1998 64 1303 1307 9546164 10.1128/aem.64.4.1303-1307.1998 PMC106145

[B33] van Dijken JP Bauer J Brambilla L Duboc P Francois JM Gancedo C Giuseppin ML Heijnen JJ Hoare M Lange HC An interlaboratory comparison of physiological and genetic properties of four *Saccharomyces cerevisiae* strains Enzyme Microb Technol 2000 26 706 714 10.1016/S0141-0229(00)00162-9 10862876

[B34] van Maris AJ Winkler AA Porro D van Dijken JP Pronk JT Homofermentative lactate production cannot sustain anaerobic growth of engineered *Saccharomyces cerevisiae*: possible consequence of energy-dependent lactate export Appl Environ Microbiol 2004 70 2898 2905 10.1128/AEM.70.5.2898-2905.2004 15128549 PMC404449

[B35] Gietz RD Woods RA Transformation of yeast by lithium acetate/single-stranded carrier DNA/polyethylene glycol method Guide to Yeast Genetics and Molecular and Cell Biology, Pt B 2002 350 87 96 10.1016/s0076-6879(02)50957-5 12073338

[B36] Verduyn C Postma E Scheffers W Van Dijken J Effect of benzoic acid on metabolic fluxes in yeasts: a continuous-culture study on the regulation of respiration and alcoholic fermentation Yeast 1992 8 501 517 10.1002/yea.320080703 1523884

[B37] Sambrook J Maniatis T Fritsch EF Molecular cloning : a laboratory manual 1989 2 Cold Spring Harbor, N.Y.: Cold Spring Harbor Laboratory

[B38] Stella CA Sáenz DA Chianelli MS Kaminszczik S A simple protocol to evaluate nitrogen utilisation in *Saccharomyces cerevisiae* Biochem Educ 2000 28 169 170 10.1016/S0307-4412(99)00139-9 10878317

[B39] Bradford MM A rapid and sensitive method for the quantitation of microgram quantities of protein utilizing the principle of protein-dye binding Anal Biochem 1976 72 248 254 10.1016/0003-2697(76)90527-3 942051

[B40] Sigma L URL: [http://www.sigmaaldrich.com]

[B41] Ellman GL Tissue sulfhydryl groups Arch Biochem Biophys 1959 82 70 77 10.1016/0003-9861(59)90090-6 13650640

