# Supplementary material for: A novel pathway to produce butanol and isobutanol in Saccharomyces cerevisiae
Source: Biotechnol Biofuels. 2013 May 4;6:68. doi: 10.1186/1754-6834-6-68 (PMC3662618; doi:10.1186/1754-6834-6-68)
Supplement: Additional file 1 — Sequence of synthesized goxB gene with codon usage optimized for Saccharomyces cerevisiae. [file 1754-6834-6-68-S1.pdf]

Additional files

Additional file 1 – Sequence of synthesized *goxB* gene with codone usage optimized for *Saccharomyces cerevisiae*

ATGAAGAAACACTACGACACTGCAGTTATAGGTGGAGGGATCATTGGTTG
TGCGATATCGTACGAATTGGCCAAAACTCAACAGAAGGTTGTCCTGCTAG
AAGCTGGAGAAGTAGGTAGAAAGACTACTAGTGCTGCTGCTGGAATGCTT
GGAGCTCATGCCGAATGCGAAAACAGGGATGCTTTCTTTGACTTTGCCAT
GCACTCACAAAGGCTTTATGAACCAGCAGGGCAAGAATTGGAAGAAGCA
TGTGGTATTGATATTAGACGTCATAATGGCGGAATGTTGAAGTTAGCCTAT
ACGGAAGAGGATATTGCCTGTTTAAGAAAGATGGATGATTTACCTAGCGT
TACCTGGTTGTCTGCTGAAGATGCATTGGAGAAGGAACCTTATGCATCGA
AAGACATACTAGGTGCATCCTTTATAAAAGATGATGTGCACGTAGAACCG
TATTATGTCTGCAAAGCCTACGCTAAAGGGGCTAGGAGATATGGTGCTGA
CATTTACGAACACACACAAGTCACCTCAGTGAAAAGAATGAACGGAGAG
TATTGCATCACAACATCAGGTGGAGATGTTTATGCCGACAAGGTTGCAGT
TGCTTCTGGTGTATGGTCTGGTCGTTTCTTTTCCCAGTTAGGTTTAGGTCAA
CCATTCTTTCCAGTAAAAGGCGAGTGTTTGAGTGTTTGGAATGACGATACC
CCATTAACCAAGACTCTTTACCATGACCATTGTTACGTGGTTCCAAGAAAG
TCCGGCAGATTGGTCATTGGTGCCACTATGAAACATGGTGATTGGTCTGAT
ACACCTGACATTGGTGGCATTGAAGCTGTGATTGGTAAGGCGAAAACGAT
GCTACCAGCAATTGAGCACATGAAAATCGATAGATTTTGGGCGGGTTTAA
GACCGGGAACAAGAGATGGCAAACCCTTCATTGGGAGACATCCCGAAGA
TAGCGGCATAATCTTTGCAGCCGGTCATTTCAGAAATGGCATACTGCTGG
CTCCTGCAACAGCTGAAATGGTCAGAGACATGATCTTGGAACGTCAGATA

AAACAAGAGTGGGAAGAGGCATTTAGGATCGATAGAAAAGAGGCGGTTC

ATATCTAA
